# Supplementary material for: Triplet Cooper pairs induced in diffusive s-wave superconductors interfaced with strongly spin-polarized magnetic insulators or half-metallic ferromagnets
Source: Sci Rep. 2017 May 16;7:1932. doi: 10.1038/s41598-017-01330-1 (PMC5434070; doi:10.1038/s41598-017-01330-1)
Supplement: Supplementary file 1 — Supplementary information for: Triplet Cooper pairs induced in diffusive s-wave superconductors interfaced with strongly spin-polarized magnetic insulators or half-metallic ferromagnets [file 41598_2017_1330_MOESM1_ESM.pdf]

Supplementary information for: Triplet Cooper pairs induced in diffusive s-wave superconductors interfaced with strongly spin-polarized magnetic insulators or half-metallic ferromagnets

Jabir Ali Ouassou, Avradeep Pal, Mark Blamire, Matthias Eschrig, & Jacob Linder

Modelling the Singh experiment

As discussed in the main article, we modelled the experiment by Singh *et al.* as a diffusive S/F/N/HM multilayer with spin-dependent tunneling boundary conditions. We set the tunneling conductance at each interface to $G_0/G = 0.4$, where G is the normal-state conductance of each material. At the S/F and F/N interfaces, we chose the modest spin-mixing conductance $G_\varphi/G_0 = 0.5$ and polarization $P = 0.2$. At the N/HM interface, however, we chose the much larger values $G_\varphi/G_0 = 10$ and $P = 0.999$. As for the magnetization directions, we assume that the magnetization of the HM is oriented along the z-axis, while the magnetization of F is along the direction $\sin\alpha\,\boldsymbol{e}_x + \cos\alpha\,\boldsymbol{e}_z$ in the xz-plane, so that α is the magnetic misalignment in the structure. In accordance with the experiment, we set the length of S to $10\xi_S$, F to $0.3\xi_S$, N to $1.0\xi_S$, and HM to $20\xi_S$, where $\xi_S = 5$ nm is the coherence length of the superconductor MoGe. However, we found no spin-valve effect $T_c(0) - T_c(\pi/2)$ for this set of parameters, where $T_c(\alpha)$ is the critical temperature for a structure with a misalignment α as defined above.

In figs. 1 to 4 below, we show that this conclusion is very robust to changes in model parameters. All plots show the critical temperature T_c measured relative to the bulk superconductor critical temperature T_{cs}. The four subfigures in each figure show the critical temperatures $T_c(0)$ and $T_c(\pi/2)$ for different magnetic configurations, as well as results for both an S/F/N/HM and S/F/N junction with the same parameters. In fact, not only do we find that $T_c(\pi/2)$ is nearly identical to $T_c(0)$ for a very wide parameter span, but we also see that the results for an S/F/N/HM and S/F/N junction look the same as well. From this, we draw the conclusion that although there is a large proximity effect in these structures – as evidenced by $T_c(0)$ and $T_c(\pi/2)$ being much smaller than T_{cs} – there is only a negligible spin-valve effect $T_c(0) - T_c(\pi/2)$. The comparison between the S/F/N/HM and S/F/N junctions show that in all cases, the observed proximity effect can be attributed solely to the F layer and not the HM layer.

The reason for this lack of spin-valve effect, seems to be that there are too many interfaces inbetween the S and HM in the S/F/N/HM junction. Since we assume that each interface in this junction is a tunneling barrier, this leads to a very low effective transparency between the S and HM layers. In other words, if all the Cooper pairs leaking out of S are either reflected at one of these interfaces, or decay inside the F, then they do not reach the HM. This, of course, means that the properties of the HM become irrelevant for the physical state of S. Thus, we end up with an effective S/F bilayer and not a spin-valve.

We therefore conclude that the the experiment by Singh *et al.* is likely to have a different mechanism than a triplet spin-valve.

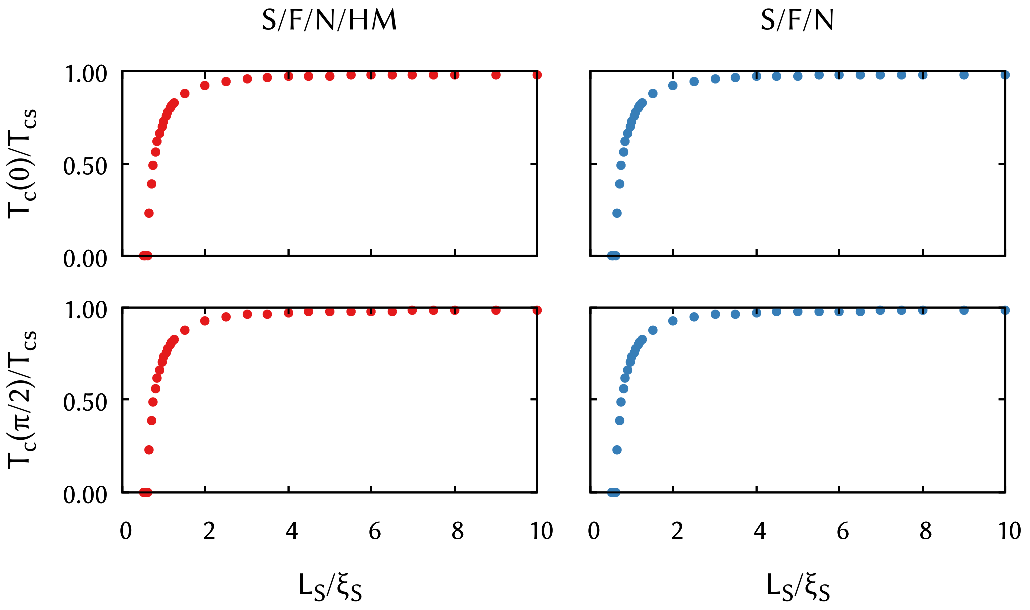

Figure 1. Critical temperature as a function of the superconductor length L_S. The other parameters were set to the experimental estimates described in the text. To see a significant proximity effect, we need the superconductor to be shorter than $\sim 2\xi_S$, which is much smaller than the $\sim 10\xi_S$ used in the experiment. This result is consistent with the conventional wisdom that the coherence length is the "healing length" of a superconductor, over which the superconducting gap is restored to nearly its bulk value: even if $\Delta \to 0$ at one end, $\Delta \to \Delta_0$ at the other end if $L_S \gg \xi_S$, resulting in a high T_c. Note that even for a superconductor as short as $1\xi_S$, we see no evidence of a spin-valve effect since the curves for $T_c(0)$ and $T_c(\pi/2)$ behave in the same way. We also see no evidence of the HM having any effect on the superconductor, since removing it produces the same T_c curves.

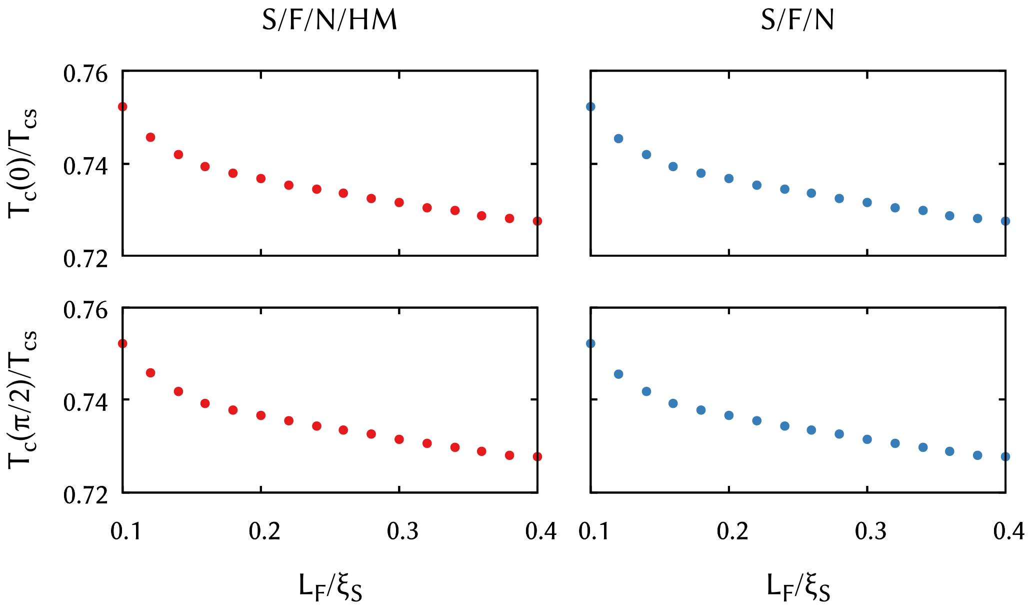

Figure 2. Critical temperature as a function of the ferromagnet length L_F. In order to see any proximity effect at all, we have chosen a superconductor length $L_S = \xi_S$ for this simulation, which is much smaller than the experimental value $10\xi_S$. The other parameters are as close to the experimental estimates as possible, as described in the text. Even with these changes, we see no evidence of a spin-valve effect even with an F length significantly different from the experimental value $0.3\xi_S$.

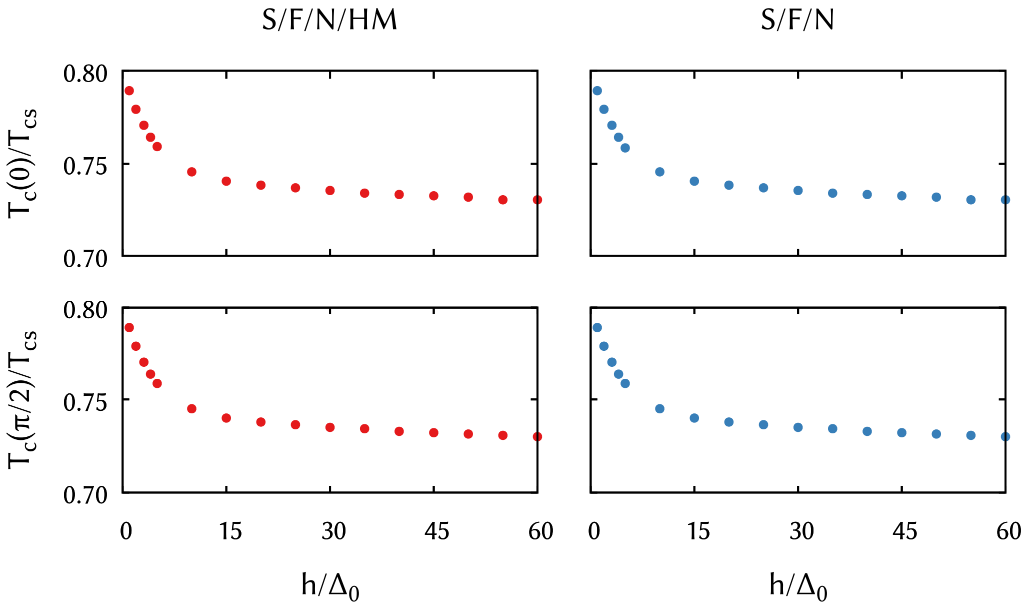

Figure 3. Critical temperature as a function of the exchange field h. In order to see any proximity effect at all, we have chosen a superconductor length $L_S = \xi_S$. The other parameters are as close to the experimental estimates as possible, as described in the text. Even if we decrease the exchange field with more than an order of magnitude compared to the value $h = 50\Delta_0$, which is already low compared to the experiment, we do not find any spin-valve effect. Increasing the exchange field would not help either, since that would cause even more Cooper pairs to decay inside the F, further reducing the number that reaches the HM.

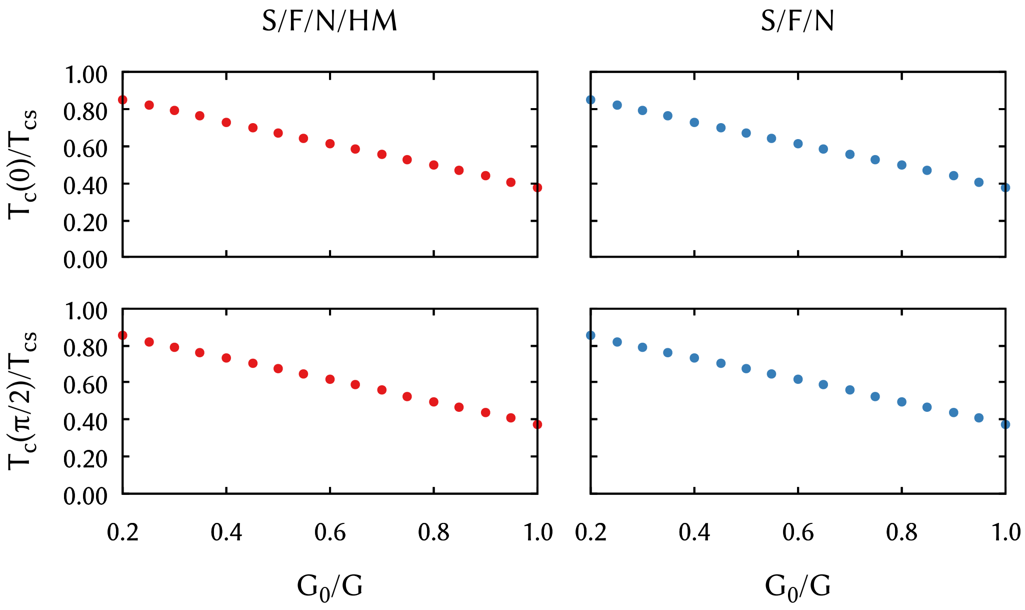

Figure 4. Critical temperature as a function of the tunneling conductance G_0/G at each interface in the junction. We chose a superconductor length $L_S = \xi_S$ for this simulation to obtain a reasonable proximity effect, but kept the other parameters as close to the experimental estimates as possible, as described in the text. Even using a relatively high tunneling conductance $G_0 = G$ does not produce any evidence of a spin-valve effect in our numerical simulations.
